# Chromatin modifiers in neurodevelopment

**DOI:** 10.3389/fnmol.2025.1551107

**Published:** 2025-05-21

**Authors:** Sarallah Rezazadeh, Hong Ji, Cecilia Giulivi

**Affiliations:** ^1^Department of Neurology, Icahn School of Medicine at Mount Sinai, New York, NY, United States; ^2^Department of Anatomy, Physiology, and Cell Biology, School of Veterinary Medicine, University of California, Davis, Davis, CA, United States; ^3^Department of Molecular Biosciences, School of Veterinary Medicine, University of California, Davis, Davis, CA, United States; ^4^UC Davis MIND Institute, Sacramento, CA, United States

**Keywords:** epigenetics, neurodevelopment, corticogenesis, DNA methylation, autism, intellectual disability

## Abstract

Emerging sequencing studies highlight the critical role of chromatin regulatory mechanisms in human diseases, particularly in neurodevelopmental and neurological disorders. Insights gained from these studies and model organism research reveal the intricate involvement of chromatin regulators in neurodevelopment, raising compelling questions about how mutations in these ubiquitous proteins drive specific dysfunctions in the nervous system. This mini review delves into key chromatin modifiers, including the histone methyl transferases NSD1 and ASH1L, the methyl-CpG-binding repressor MeCP2, and the enzymatic repressor EZH2. While functions of these proteins are relatively well-studied, the roles of many other chromatin modifiers in neurodevelopment remain poorly understood. Existing therapies targeting chromatin modifiers have shown promise, with some achieving significant clinical success. The possibility that neurological dysfunctions may be treatable even later in life underscores the urgency of prioritizing chromatin modifiers as therapeutic targets. In this mini review, we critically evaluate the current understanding of chromatin modifiers, focusing on methylation, and spotlight their pivotal roles in early brain development and neurological disorders. By advancing this field, we aim to inspire progress toward innovative treatments for these challenging conditions.

## 1 Introduction

Neurodevelopmental Disorders (NDDs) include a broad spectrum of conditions that emerge during the development of the central nervous system (CNS) (Mossink et al., 2021; Parenti et al., 2020). These disorders usually begin in early childhood or teen years and lead to varying degrees of neuropsychiatric impairment, often accompanied by a wide range of symptoms. In this context, several neuropsychiatric and neurological disorders—such as autism spectrum disorders (ASD), Tourette syndrome (TS), schizophrenia (SCZ), and bipolar disorder—are recognized as resulting from impaired CNS development. Combining these symptoms and their underlying causes poses challenges for diagnosis and treatment.

Recent studies have shown that between 5 and 10% of Mendelian disorders result from variants in genes encoding factors that are directly associated with the epigenetic machinery. These disorders, characterized by genome-wide alterations in chromatin fiber organization, are known as chromatinopathies. Approximately 75% of these disorders are associated with neurodevelopmental issues. Studies in model organisms and humans have demonstrated that this group of genes is dosage-sensitive, with haploinsufficiency serving as the primary disease mechanism (Fahrner and Bjornsson, 2019).

**Figure 1:**
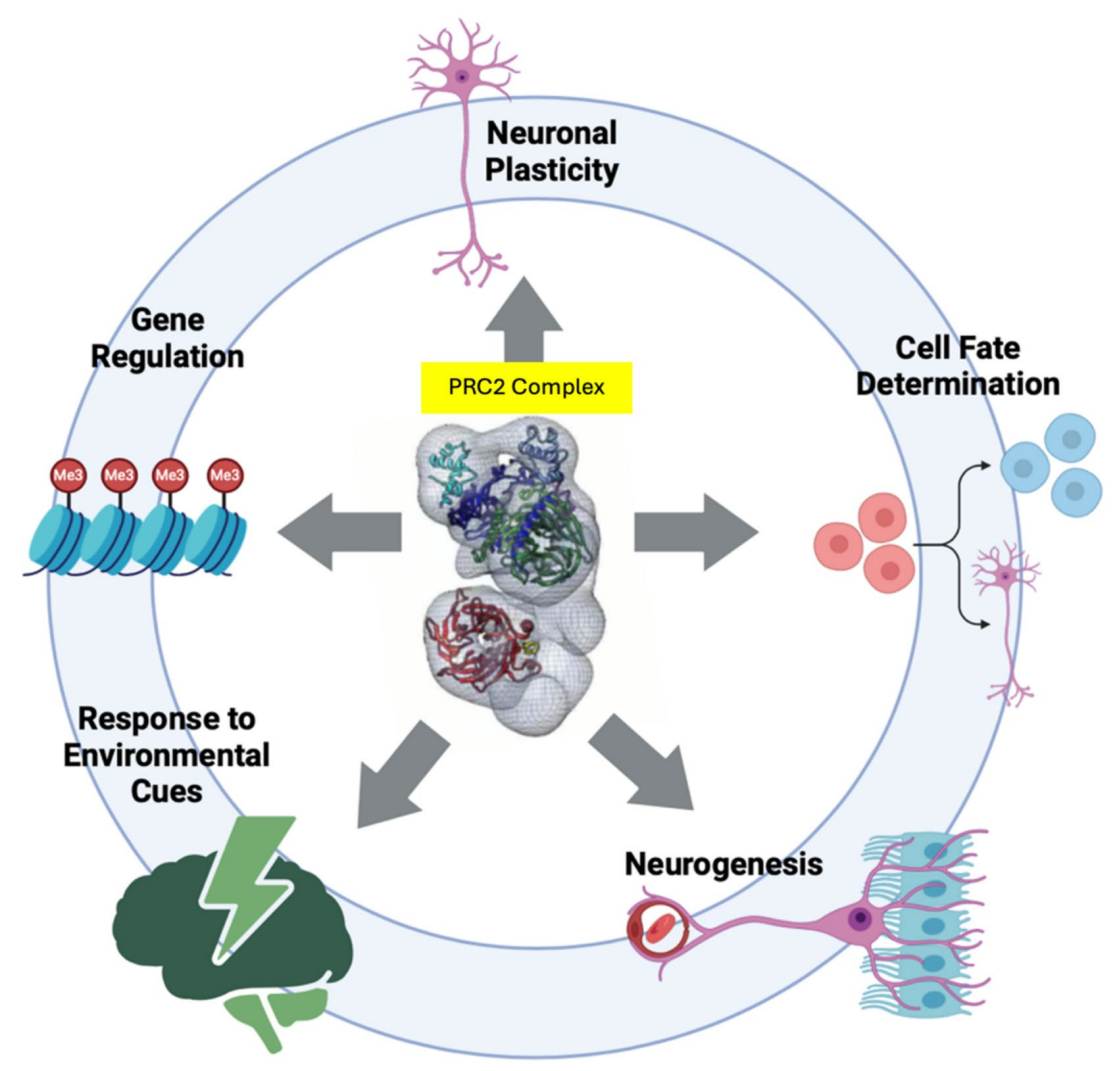
Chromatin modifying enzymes play several critical roles in neurodevelopment by regulating Gene Regulation, Neuronal Plasticity, Cell Fate Determination, Response to Environmental Cues, and Neurogenesis. Depicted at the center of circle is PRC2 complex (Ciferri et al., 2012) which represents chromatin modifiers. Illustration made by BioRender.

Chromatin-modifying enzymes are proteins that alter the structure and function of chromatin by adding (writing) or removing (erasing) chemical modifications to the histone proteins or to the DNA itself (Chen and Dent, 2014). These modifications are crucial for satisfying cellular needs, such as gene expression and DNA repair, by affecting chromatin accessibility and altering the physical interaction between transcriptional regulatory elements and histones (Chen and Dent, 2014). This facilitates or impedes the recruitment of transcriptional factors on regulatory elements, which could result in transcriptional activation and repression, respectively. Chromatin-modifying enzymes play several critical roles in neurodevelopment, such as regulating gene regulation, neuronal plasticity (Herre and Korb, 2019; Singh et al., 2015), cell fate determination (Aughey, 2023), response to environmental cues (Fang et al., 2014; Turner, 2009), and neurogenesis (Park et al., 2022; Salinas et al., 2020; Lossi et al., 2024) (Figure 1).

An increasing body of evidence has explored the characterization of chromatin modifiers and their role in neurodevelopment (Niklison-Chirou et al., 2020; Mitrousis et al., 2015; Lee and Lee, 2010; Zhang et al., 2020). However, it is essential to emphasize the need for a more comprehensive and precise understanding of these modifiers. This review will delve into our evolving understanding of how chromatin modifications, specifically histone and DNA methylation impact early brain development. We will provide an overview of the current knowledge regarding their regulatory roles in human brain development, primarily derived from studies on the impaired activity of chromatin modifiers in neurodevelopmental disorders.

Chromatin Modifications in Early Brain Development

Next,-generation sequencing has advanced the study of these NDDs by identifying 750 genes believed to be causative of NDD (Kochinke et al., 2016). Notably, many of these NDDs are considered monogenic disorders, although the pathological mechanism driving these NDDs is mainly unknown. Interestingly, epigenetic factors account for 8% of neurodegenerative diseases and are increasingly being acknowledged as significant contributors to neurological disorders (Kleefstra et al., 2014). Mutations in genes related to DNA methylation, modifications of histone proteins (such as methylation, acetylation, and phosphorylation), and ATP-dependent chromatin remodeling have been linked to the development of NDDs, resulting in a range of phenotypes from mild to severe (Mossink et al., 2021).

The cerebral cortex, the neocortex, is the brain region primarily responsible for abstract thinking and the distinct cognitive abilities that define humans (Lourenco et al., 2020; Wang et al., 2025). It is the most complex biological structure and develops during embryonic growth through a series of genetically determined molecular and cellular events. This developmental process mirrors the evolutionary emergence of the human cerebral cortex (Cadwell et al., 2019). The fundamental methods of neocortical development involve the proliferation of neural stem and progenitor cells (NSPCs), the generation and migration of neurons from their origin to their final positions within the neocortical layers, as well as the growth of neural processes and the formation of nerve connections (Cadwell et al., 2019; Diaz and Gleeson, 2009; Mannens et al., 2024).

**Figure 2:**
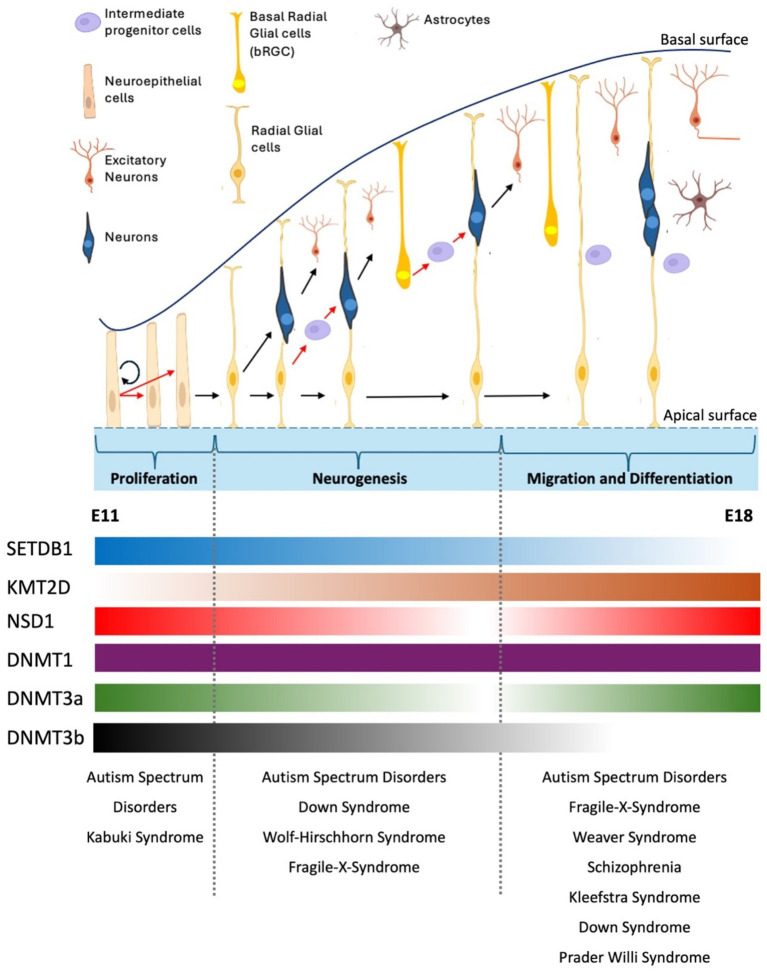
Overview of cortical neurogenesis in mouse brain development. During early cortical neurogenesis, neuroepithelial cells (NEs) initially undergo symmetric divisions to expand their population, followed by asymmetric divisions that produce apical neural progenitor cells (NPCs), including radial glial cells (RGs) in the ventricular zone (VZ) and pioneer neurons (Qian et al., 2000; La Manno et al., 2021). The neuronal population is expanded through indirect neurogenesis, which involves the generation of neuron-amplifying cells like intermediate progenitor cells (IPCs) and basal radial glial cells (bRGCs) from RGCs. Newly formed neurons migrate either through soma translocation or locomotion, utilizing the slender fibers of RGCs as guidance to exit the ventricular/subventricular zone (VZ/SVZ), pass through the intermediate zone (IZ), and reach their destination in the cortical plate (CP). During migration and differentiation stage, RGCs give rise to or transition into glial cells, including astrocytes, oligodendrocytes, or oligodendrocyte precursor cells (Sokpor et al., 2022). Shown below the diagram the dynamic mRNA expression of some chromatin modifiers. Whereas DNMT1 shows stable expression during mouse embryonic brain development other modifiers such as SETDB1, KMT2D show significant changes in their gene expression. Dnmt3b, for instance, shows robust expression in SVZ at early proliferation stage, but its expression gradually diminishes and becomes undetectable at E15 (Yao et al., 2016). Neurological disorders that arise due to perturbation of stages of brain development are shown (See text for details). RNA expression profile obtained from Allen Brain Atlas. E11, mouse embryonic day 11. E18, mouse embryonic day 18.

The human cortex relies on two waves of cell proliferation. Initially, polarized neuroepithelial cells (NECs), which line the lumen of the neural tube, undergo symmetrical divisions through which the progenitor pool is increased and finally elongate in cell shape, becoming radial glial cells (RGCs) (Cadwell et al., 2019) (Figure 2). RGCs can gradually undergo differentiative divisions to generate neurons directly (direct neurogenesis). This phase, occurring in the ventricular and subventricular regions, expands the progenitor pool, leading to the enlargement of the subventricular zone (SVZ) in the cortex and the subgranular zone (SGZ) in the hippocampus (Lui et al., 2011). Subsequently, an asymmetrical division phase influences the number of neurons in the various cortical layers (Florio and Huttner, 2014) (Figure 2). In this case, RGCs generate intermediate progenitors (IPs) that further divide to generate neurons (indirect neurogenesis). Whereas direct neurogenesis is predominant at the onset of corticogenesis, later in development, indirect neurogenesis is more essential and critical to generating the bulk of cortical neurons (Cadwell et al., 2019; Lui et al., 2011).

Migration is crucial in bringing together projection neurons (PNs) and interneurons (INs) within the cerebral cortex (Silva et al., 2018). This process facilitates the organization of these diverse cell types and influences the overall functionality of cortical circuits (Florio and Huttner, 2014). PNs migrate radially within the cortical wall, whereas INs originate from the ventral forebrain and reach the cortex by moving along tangential paths guided by various spatially and temporally expressed chemo-attractive and -repellent signaling molecules (Florio and Huttner, 2014; Silva et al., 2018). Besides the effects of the local extracellular signals, multiple factors in neuronal migration, fate determination, and morphological maturation depend on intrinsic transcriptional networks, as shown by various fate mapping and clonal analysis studies (Florio and Huttner, 2014).

Most common histone modifications include Acetylation, Methylation, Phosphorylation, and Ubiquitination. The epigenetics of neural development are challenging to catalog and understand due to the vast number of events and cell types involved. Here, we discuss DNA and histone methylation with focus on early brain development.

### 1.1 Histone and DNA methylations in early brain development

#### 1.1.1 Control of embryonic neurogenesis by DNA methylation

DNA methylation, primarily involving adding methyl groups to the 5-carbon of cytosine residues within CpG dinucleotides, exerts its regulatory influence on transcription through at least two mechanisms (Hyun et al., 2017). Firstly, the methylation of CpG dinucleotides alters DNA structure, directly preventing the binding of methylation-sensitive transcriptional activators. Secondly, and more broadly, 5mC can be recognized and interpreted by a group of ‘readers’ known as methyl-CpG-binding proteins, which can then recruit chromatin remodeling complexes. Among these, proteins containing a methyl-binding domain (MBD), particularly MBD1 and MeCP2, are well-studied (Kinde et al., 2016; Tillotson and Bird, 2020). This effectively silences gene expression, which is essential for turning off genes that are not required in specific neuronal subtypes or developmental stages.

Beyond promoter regions, DNA methylation occurs in gene bodies, intragenic regions, and intergenic regions, where it plays additional roles in regulating genomic function (Kinde et al., 2016). Furthermore, DNA methylation within intergenic regions and repetitive elements helps maintain genome stability by repressing the activity of transposable elements, which could otherwise disrupt neuronal genome integrity (Shenker and Flanagan, 2012; Pappalardo and Barra, 2021). In addition to these repressive functions, DNA methylation can modulate alternative promoter choice, enabling the selective activation or silencing of specific transcript variants from the same gene (Maunakea et al., 2013; Neri et al., 2017). These changes in gene body DNA methylation patterns could be interesting given the dramatic transcriptional plasticity occurring in the brain at early development (Colantuoni et al., 2011).

Human fetal brain development is marked by extensive alterations in DNA methylation pattern (Spiers et al., 2015). The importance of CG methylation in neurogenesis could be summarized into 3 stages: Proliferation, Differentiation, and Maturation. During early gestation, neural stem cells (NSCs) lack multipotentiality and differentiate exclusively into neurons (Obernier and Alvarez-Buylla, 2019). As gestation progresses, NSCs gradually gain multipotentiality and begin differentiating into astrocytes and oligodendrocytes in late gestation (Temple, 2001). In a study, Stadler et al. uncovered the coordination between transcription factors and DNA methylation changes that drive the generation of NPCs (Stadler et al., 2011). This work identified low-methylated regions (LMRs) with an average methylation of 30%, which were CG-poor distal regulatory regions (Stadler et al., 2011). Interestingly, these NP-specific LMRs were enriched with motifs from various neuronal transcription factors, including Sox17 and Pax6, which could activate the expression of genes crucial for neuronal development (Stadler et al., 2011). Loss of DNA methylation on *Hoxa* gene cluster, which is implicated in neural crest development, and *Gata2* and *Pax6*, which are implicated in neurogenesis, on the other hand, due to deletion of the histone deacetylase sirtuin 6 (SIRT6), results in skewed development toward neuroectoderm (Etchegaray et al., 2015). These studies highlight how the precise regulation of locus-specific transcriptional programming can influence neurogenesis early in development.

Besides, of NSCs and NPSc maintenance, DNA methylation also contributes to differentiation stage during embryonic neurogenesis. During early brain development, methylation of the CG sites within the STAT3 binding region, for instance, can prevent the binding of activated STAT3 to the GFAP (Glial Fibrillary Acidic Protein) promoter, thus, hindering astrogliogenesis (Fan et al., 2005).

Intriguingly, in addition to cell type-specific methylation patterns, there are also sex differences and unique sex-specific DNA methylome alterations during human brain development, including at autosomal sites (Spiers et al., 2015). The highest-ranked CG site that shows a sex-specific neurodevelopmental trajectory is located in the RBPJ gene, which encodes a transcriptional regulator in the Notch signaling pathway and plays a direct role in neurogenesis and neuronal maturation (Spiers et al., 2015; Arevalo et al., 2011; Xu et al., 2014; Lampada and Taylor, 2023).

In contrast, non-CG methylation, particularly methylation at cytosine-adenine (CA) dinucleotides (mCA), is a distinctive feature of stem cells (Tan et al., 2019; Lee et al., 2017) and neurons in the adult brain (Guo et al., 2014). Unlike mCG, ubiquitous across most cell types, mCA emerges primarily in postmitotic neurons during development and accumulates throughout life. This neuron-specific epigenetic mark is believed to play a unique role in neuronal function, including regulating activity-dependent gene expression and synaptic plasticity (Guo et al., 2014; de Mendoza et al., 2021).

The deposition of mCA is facilitated by DNA methyltransferase 3A (DNMT3A), which is highly expressed in neurons during critical periods of neurogenesis (Chen and Chan, 2014; Gabel et al., 2015). The accumulation of mCA is often enriched in regions outside of traditional promoter CpG islands, such as gene bodies and distal regulatory elements (Clemens et al., 2020). It has been shown to influence transcriptional activity in unique ways compared to mCG. For instance, mCA appears to correlate with long-term gene repression, potentially by recruiting specific methyl-CpG-binding proteins like MeCP2, which can modulate chromatin states and neuronal gene expression (Stroud et al., 2017). This then may have a long-term effect on gene expression and neuronal function much later in life. These findings seem to align with the observation that mCG is susceptible to oxidation by TET proteins, leading to its active removal (Pastor et al., 2013).

The presence of mCA in neurons reflects the specialized demands of the nervous system, where epigenetic mechanisms must support complex, long-term processes such as learning, memory, and adaptation to environmental stimuli (Stroud et al., 2017). The distribution and function of mCA in the neuronal genome underscore its importance in fine-tuning transcriptional programs essential for neural circuit formation and maintenance. Another difference is unlike mCG, which is consistently high across chromosomes, mCA levels can vary substantially in megabase-scale regions of the genome. This “regional mCA” is organized partly by topologically associating domains (TADs) of chromatin folding. Genes and enhancers located within TADs with a high mCA “set point” will generally exhibit elevated mCA levels in the mature brain, unlike those found in TADs with lower mCA set points (Hamagami et al., 2023). Deposited by DNMT3A, global levels of mCA vary across different brain regions and cell types, and patterns of mCA across genes display strikingly cell-type-specific patterning (Sharma et al., 2016; Mo et al., 2015).

Despite significant progress in understanding DNA methylation, several fundamental questions remain regarding developmental methylation turnover mechanisms, particularly in specific promoters such as *HOX* and *STAT*. These genes are critical for development and cellular differentiation, and their methylation status can influence gene expression patterns that persist throughout an organism’s life. However, the precise mechanisms by which these methylation marks are added, maintained, or removed are still being explored.

One of the key questions is whether demethylation occurs through a passive or active process. Passive demethylation typically occurs when DNA methylation is not maintained during cell division. This process depends on DNA replication, where the failure of DNA methyltransferases (such as DNMT1) to restore methyl marks to the newly synthesized DNA strand leads to a gradual dilution of methylation over successive cell divisions. This mechanism is often observed in early embryonic development when global epigenetic reprogramming occurs.

In contrast, active demethylation involves enzymatic processes that remove methyl groups independently of DNA replication. This can occur through oxidation-mediated pathways involving ten-eleven translocation (TET) enzymes, which convert 5-methylcytosine (5mC) into intermediates such as 5-hydroxymethylcytosine (5hmC), ultimately leading to base excision repair and replacement with unmethylated cytosine. Such active processes suggest that demethylation may be dynamically regulated in response to cellular or environmental signals.

Another crucial aspect of methylation turnover is whether metabolites regulate it. DNA methylation and demethylation are intimately linked to metabolic pathways. For instance, the availability of S-adenosylmethionine (SAM), the universal methyl donor for DNA methylation, depends on cellular metabolism, particularly folate and methionine cycles. Similarly, TET enzymes require *α*-ketoglutarate, a metabolite derived from the Krebs cycle, for their demethylation activity. This metabolic dependence suggests that changes in nutrient availability, oxidative stress, or energy status could influence methylation dynamics.

Furthermore, growing evidence shows that life experiences may influence methylation turnover during adulthood. Environmental factors such as stress, diet, toxins, and social interactions have been shown to induce epigenetic changes, including alterations in DNA methylation patterns. For example, exposure to chronic stress has been linked to changes in the methylation of genes involved in stress response pathways, which can have long-term effects on gene expression and behavior. Similarly, diet-induced shifts in one-carbon metabolism can affect methylation patterns, potentially influencing disease susceptibility or aging processes.

While significant strides have been made in DNA methylation, fundamental questions remain about the precise mechanisms governing methylation turnover, particularly in critical gene promoters like *HOX* and *STAT*. Understanding whether demethylation occurs passively or actively, how metabolic factors contribute to methylation regulation, and whether life experiences can reshape these epigenetic marks will be essential for unraveling the complexities of gene regulation and its implications for development, disease, and aging.

#### 1.1.2 Control of embryonic neurogenesis by histone methylation

Histone methylation occurs on both arginine and lysine residues and is essential for regulating transcription (Allfrey and Mirsky, 1964). Methylation is more complex than acetylation, which operates as a binary system (acetylation or no acetylation). There are two classes of lysine methyltransferases (KMT) in humans, and this classification depends on the presence or absence of the catalytic Su(var)3–9, E(z) (enhancer of zeste), and trithorax (SET) domain (Greer and Shi, 2012; Nguyen and Zhang, 2011). Similar to acetylation, methylation is removed by a group of enzymes. These enzymes, called lysine demethylases (KDMs) (Greer and Shi, 2012) are classified into two types based on their catalytic domains: those with a flavin adenine nucleotide (FAD)-dependent oxidase domain and those with a Jumonji C (JmjC) domain (Shi and Whetstine, 2007).

Common histone methylation includes adding one or multiple methyl groups to lysine 36, 27, 9 and 4 of histone tails, which have distinct roles in gene regulation, often working in conjunction with DNA methylation. Altered mCA levels or distribution have been implicated in neurodevelopmental and neurological disorders, including Rett syndrome and autism spectrum disorders, highlighting its significance in maintaining neuronal health and function. This unique feature of neuronal epigenetics continues to be a topic of active research, offering insights into the specialized regulatory landscapes of the brain. Interestingly, it was shown that regional and gene expression-associated mCA patterns depend on H3K36me2 levels (Hamagami et al., 2023). Specifically, H3K36me2 is required for TAD-scale DNMT3A targeting and mCA deposition in postmitotic neurons (Hamagami et al., 2023; Weinberg et al., 2019). H3K36me2 is deposited across the neuronal genome in the developing nervous system through NSD1 (an H3K36 methyltransferase). However, it is likely that other H3K36 methyltransferases, including ASH1L and NSD2, can also deposit and maintain this mark (Zaghi et al., 2019). Once deposited, SETD2 converts H3K36me2 to H3K36me3 in a transcription-dependent manner (Edmunds et al., 2008). In general, post-translational modifications of H3K36 (H3K36me2 and me3) have an extensive role in multiple cellular processes such as (i) transcriptional fidelity (initiation, elongation, and exon inclusion/exclusion) (Sessa et al., 2019), (ii) DNA methylation, (iii) and DNA repair (Wagner and Carpenter, 2012; Rezazadeh et al., 2020). Interestingly, mutations in genes encoding for the factors that write or read H3K36 methylation, such as NSD1, ASH1L, SETD2, and NSD2, have been linked to NDD with autistic features or cognitive deficits, further stressing the importance of H3K36 metabolism in neurodevelopment (Wilson et al., 2022).

H3K36me2/3 in *cis* on the same or opposite histone tail inhibits Polycomb Repressive Complex 2 (PRC2) catalytic activity adding methyl groups onto H3K27 (Yuan et al., 2011). This general exclusivity of H3K27me3 and H3K36me2/3 on the genome is vital in setting up developmental programs and ensuring neural transcriptional identity (Schmitges et al., 2011; Alabert et al., 2020). A compelling line of evidence underscoring the critical role of H3K27 methylation in early brain development arises from studies on Weaver syndrome (WVS; OMIM 277590). WVS is a rare autosomal dominant disorder resulting from mutations in the genes encoding subunits of the PRC2, including EZH2, the catalytic subunit responsible for depositing the H3K27me3 mark (Tatton-Brown et al., 2013). This post-translational modification is crucial for establishing repressive chromatin states and silencing developmental genes in a tightly controlled manner during embryogenesis and subsequent tissue-specific development (Hansen et al., 2008). Weaver syndrome is characterized by a constellation of features, including generalized overgrowth, craniofacial dysmorphism, advanced bone age, and intellectual disability (Tatton-Brown et al., 2013; Cohen et al., 2016; Gao et al., 2024; Gibson et al., 2012). These phenotypic hallmarks indicate a disruption in the finely tuned epigenetic regulation of genes required for proper growth and neurodevelopment. Mutations in PRC2 subunits, such as EZH2, compromise the complex’s ability to effectively catalyze H3K27 trimethylation, leading to dysregulation of gene silencing (Margueron et al., 2009). In the context of neurodevelopment, aberrant H3K27 methylation due to functional perturbation of the H3K27 methyltransferase Ezh2, leads to defects in neuronal differentiation (Hahn et al., 2013; Ritchie and Lizarraga, 2023).

Beyond intellectual disability, the overgrowth phenotype of WVS further illustrates how epigenetic regulation via PRC2 influences developmental pathways across multiple organ systems. Since PRC2 represses proliferation-promoting genes during early development, mutations that reduce its activity can lead to unchecked cellular growth, as observed in Weaver syndrome. Interestingly, animal models of WVS show abnormal neurogenesis in the cerebral cortex, cerebellum, and spinal cord during nervous system development. Moreover, alteration of neurogenesis induced by haploinsufficiency in PRC2 catalytic subunit EZH2 has also been associated with various neurogenic processes, such as the reduced NPCs pool, changes in neural cell fate as well as in neural migration pattern (Di Meglio et al., 2013; Akizu et al., 2016; Zhang et al., 2014; Zhang et al., 2015; Hirabayashi et al., 2009; Hwang et al., 2014). This example underscores the broader importance of H3K27 methylation as a regulatory mechanism in the neurogenesis. Studies on WVS provide valuable insights into how disruptions in the balance of histone methylation contribute to complex human disorders, emphasizing the therapeutic potential of targeting epigenetic pathways in such conditions (Suhr and Rottinger, 1986).

Like H3K27me3, methylations on H3K9 also mark silenced genes. Members of the SET domain-containing histone methyltransferase family catalyze this modification. Suv39h1 and Suv39h2 (also known as KMT1A/1B) catalyze H3K9me2 and H3K9me3, SETDB1 also catalyzes H3K9me2 and H3K9me3, while SETDB2 specifically introduces H3K9me3. Additionally, G9a and GLP and G9a (EHMT1 and EHMT2) catalyze H3K9me1 and H3K9me2 (Padeken et al., 2022; Xiong et al., 2017). Abnormal methylation of H3K9, caused by genetic variants in histone methyltransferases, is associated with neurodevelopmental and behavioral disorders. A prominent example comes from studies of Kleefstra syndrome (KS; OMIM 610253), which is characterized by intellectual disability, childhood hypotonia, and autistic-like features. KS pathology has been linked to mutations in EHMT1, a gene that plays a role in cortical neuronal network development and homeostatic plasticity (Benevento et al., 2016). Consistent with the above, genetic knockdown of EHMT1 prevented the increase of H3K9me2 and synaptic scaling up, suggesting that H3K9me2-mediated changes in chromatin structure monitor a repressive transcriptional program that controls cortical neuronal network development (Benevento et al., 2016; Balan et al., 2021).

In addition to its well-established role in fine-tuning cortical synaptic plasticity, recent investigations have uncovered a novel and pivotal role for H3K9 methylation in the regulation of embryonic neurogenesis (Guerra et al., 2021; Kunoh et al., 2024; Tan et al., 2012). Specifically, during embryonic neurogenesis, the deletion of SETDB1 results in decreased H3K9 trimethylation and disrupted gene expression, including Sox9 and IAP, causing significant impairment of early neurogenesis. SETDB1 deficiency leads to an increase in neurons in the deep layers and a decrease in neurons in the upper layers, indicating that SETDB1 is essential for the proper neuronal composition and distribution across all six cortical layers (Tan et al., 2012).

Below, we explore the specific roles of H3K9 methylations in NDDs:

Rett Syndrome, a neurodevelopmental disorder primarily affecting females, is caused by mutations in the *MECP2* gene, which encodes a DNA methyl-binding protein (Amir et al., 1999; Chahrour and Zoghbi, 2007; Pietrobono et al., 2005). MECP2 regulates transcription by interacting with various chromatin modifiers, including SUV39H1, a key methyltransferase responsible for adding H3K9me3 (Tillotson and Bird, 2020). In Rett syndrome, the loss of functional MECP2 disrupts the normal silencing of genes by H3K9 methylation (Tillotson and Bird, 2020). This leads to activating genes that should remain silent, such as those involved in neuronal differentiation and synaptic function. This improper regulation of H3K9me3 contributes to the cognitive decline, motor dysfunction, and developmental regression characteristic of the disease (Tillotson and Bird, 2020).

In Fragile X Syndrome, the *FMR1* gene is silenced due to an expansion of CGG repeats, a process regulated by DNA methylation and H3K9 methylation (Liu et al., 2018; Kumari and Usdin, 2010). EHMT1 and EHMT2, histone methyltransferases responsible for H3K9me2, are involved in silencing the *FMR1* gene. In Fragile X syndrome, this silencing is abnormally maintained through H3K9 and DNA methylation, leading to the loss of fragile X messenger ribonucleoprotein (FMRP) (Kumari and Usdin, 2010; Eiges et al., 2007). The absence of FMRP disrupts synaptic plasticity, leading to intellectual disabilities, social and communication impairments, and other neurodevelopmental issues seen in individuals with Fragile X syndrome (Brouwer et al., 2009; Willemsen et al., 2004; Darnell et al., 2001; Willemsen et al., 2011).

In Angelman Syndrome, a neurodevelopmental disorder characterized by developmental delay, intellectual disability, seizures, and motor dysfunction, the UBE3A gene is typically inactivated in neurons through imprinting (Buiting et al., 2016; Nicholls and Knepper, 2001). In individuals with Angelman Syndrome, the paternal allele of the UBE3A gene is silenced, and the loss of function of the maternal allele leads to the disease (Buiting et al., 2016). Research has shown that H3K9 methylation is involved in silencing the paternal UBE3A allele in neurons, and disruption of the enzymes responsible for this modification can lead to improper silencing of the gene (Benevento et al., 2016; LaSalle et al., 2015; Kleefstra et al., 2006; Kleefstra et al., 2012; Martens et al., 2016).

Prader-Willi Syndrome (PWS) is another neurodevelopmental disorder caused by the loss of paternal gene expression due to a deletion or silencing of the region on chromosome 15 that contains imprinted genes (Nicholls and Knepper, 2001). Like Angelman Syndrome, the H3K9 methylation mark is involved in silencing the paternal alleles of the imprinted genes, and disruption of this process can lead to PWS (LaSalle et al., 2015). Studies have shown that SUV39H1 and EHMT2 play essential roles in regulating this silencing, and their dysregulation can contribute to developmental delays, cognitive impairments, and other features characteristic of PWS, such as hyperphagia and obesity (Deimling et al., 2017; Xin et al., 2003; Kim et al., 2017).

H3K9 methylation is also implicated in other neurodevelopmental disorders, including schizophrenia, and intellectual disabilities, in which altered gene expression due to the improper regulation of chromatin structure leads to abnormal neuronal function and development (Kleefstra et al., 2014). In these conditions, mutations or dysregulation of H3K9 methyltransferases such as SUV39H1 and EHMT2 have been found to impair transcriptional repression, leading to the dysregulation of genes critical for synaptic plasticity, neuronal differentiation, and other key processes involved in brain development (Padeken et al., 2022; Benevento et al., 2016; Balan et al., 2021; Guerra et al., 2021; Martens et al., 2016; Balemans et al., 2013).

H3K4 trimethylation is commonly found at the promoter regions of actively transcribed genes, serving as a signal for the recruitment of transcriptional machinery, including RNA polymerase II and various transcription factors (Bannister and Kouzarides, 2011). This mark not only enhances the accessibility of chromatin but also facilitates the assembly of the transcriptional pre-initiation complex, thus promoting the initiation of gene expression (Bannister and Kouzarides, 2011). The presence of H3K4me3 is often coupled with removing repressive marks such as H3K27me3, creating a dynamic and context-dependent environment for gene expression (Flanagan et al., 2005; Balasubramanian et al., 2012; Akkers et al., 2009). Moreover, enzymes like SET1 and MLL complex, responsible for depositing H3K4 methylation, play essential roles in regulating both development and differentiation by controlling the expression of key regulatory genes (Greer and Shi, 2012; Gregory et al., 2007). During embryonic neurogenesis, SETD1A is enriched on the *β-catenin* promoter by histone cell cycle regulator (HIRA), a histone chaperone, leading to an increase in H3K4me3 levels, resulting in the induction of *β-catenin* expression in mouse embryonic NSCs. These facilitate neurogenesis of the neocortex (Li and Jiao, 2017). Heterozygous mutations in this gene are associated with two conditions: epilepsy, early onset with or without developmental delay (OMIM 618832), and neurodevelopmental disorder with speech impairment and dysmorphic facies (OMIM 619056). Heterozygous mutations in *SETD1B* gene or microdeletions of chromosome 12q24 are associated with intellectual developmental disorders with seizures and language delay (OMIM 619000).

The importance of lysine 4 methylation on histone H3 (H3K4me) in human brain development is highlighted by its remarkable locus-specific redistribution in prefrontal cortex (PFC) neurons of individuals affected by ASD (Cheung et al., 2010; Shulha et al., 2012). A recent hallmark study conducted on individuals aged 0.5 to 69 years identified approximately 6,000 genes that are highly enriched in the H3K4me3 mark in neurons compared to non-neuronal cells of the PFC. This study emphasizes the age- and cell type-related epigenomic changes, including an excessive modification of H3K4me3 at developmental genes in the prefrontal cortex (PFC) neurons of newborns, which gradually diminishes after birth (Cheung et al., 2010). The metabolism of H3K4me2/3 is also frequently impaired in X-linked intellectual disability (XLID) patients. Consistent with this, disruption of the mouse gene involved in H3K4 methylation turnover recapitulates adaptive and cognitive abnormalities, including impaired social behavior, memory deficits, and aggression (Iwase et al., 2016), suggesting that impaired H3K4me3 turnover is causal to XLID.

As highlighted throughout this discussion, the pathogenesis of numerous neurodevelopmental disorders is intricately tied to disruptions in histone methylation patterns, with many of these changes arising from loss-of-function mutations in key regulatory factors. These modifications are not merely static alterations but dynamic processes crucial for the proper development and functioning of the nervous system. The enzymes responsible for adding (writers) and removing (erasers) these methylation marks play fundamental roles in orchestrating the precise regulation of gene expression during neurogenesis, neuronal differentiation, and synaptic plasticity (Zaghi et al., 2019; Wilson et al., 2022). So far, we have examined key DNA and histone methylation patterns and their importance in early brain development. In the next section, we will delve further into the essential chromatin factors that play a role in histone methylation turnover. We will investigate how their dysregulation contributes to neurodevelopmental disorders and emphasize their potential as therapeutic targets for future interventions. We will discuss two crucial aspects: firstly, the role of histone methyltransferases (KMTs) in neurodevelopment, and secondly, the function of histone and DNA demethylases, as well as the implications of their dysregulation.

#### 1.1.3 Histone methyl transferases

##### 1.1.3.1 H3K4 methyltransferases

Histone 3 lysine 4 methyltransferases (often abbreviated as H3K4 methyltransferases) are enzymes responsible for adding methyl groups to the lysine 4 residue of histone protein H3, a key modification in the activation of gene expression (Shilatifard, 2008). Specifically, these methyltransferases catalyze the transfer of methyl groups from *S*-adenosyl methionine (SAM) to the *ε*-amino group of the lysine 4 (K4) side chain on histone H3 (Hyun et al., 2017). The main types of H3K4 methyltransferases can be broadly classified into SET-domain-containing proteins (see below) and non-SET domain proteins (Hyun et al., 2017).

##### 1.1.3.2 The COMPASS complex

The COMPASS (Complex of Proteins Associated with Set1) complex is a highly conserved and well-studied group of histone methyltransferases found in eukaryotic organisms, where it plays a crucial role in the regulation of gene expression through the methylation of histone H3 at lysine 4 (H3K4) (Allis et al., 2007), primarily adding di- and trimethyl groups to the lysine residue (Eissenberg and Shilatifard, 2010; Shilatifard, 2012).

At the heart of the COMPASS complex is SET1, which exists in two isoforms, Set1A and Set1B, and serves as the catalytic core of the complex. These SET1 isoforms contain a conserved SET domain responsible for transferring methyl groups onto H3K4 (Shilatifard, 2012). The activity of SET1 is finely regulated by its interactions with various other subunits within the complex, as well as by post-translational modifications. In addition to SET1, the COMPASS complex includes several other key components, such as WDR5, RBBP5, ASH2L, and DPY30, each of which plays a vital role in stabilizing the complex, facilitating its binding to chromatin, and enhancing the specificity of H3K4 methylation at specific genomic regions (Shilatifard, 2012).

The COMPASS complex is essential for establishing proper transcriptional programs, particularly during development, differentiation, and cellular response to environmental cues (Eissenberg and Shilatifard, 2010; Cenik and Shilatifard, 2021). It maintains the transcriptional activation of housekeeping genes and regulates the expression of genes required for specific developmental processes, including neuronal differentiation and synaptic plasticity in the nervous system (Eissenberg and Shilatifard, 2010) (Tables 1, 2). Additionally, the precise regulation of H3K4 methylation by COMPASS is vital for maintaining cellular identity and genomic stability, as improper methylation patterns can lead to aberrant gene expression and contribute to diseases such as cancer and neurodevelopmental disorders (Shilatifard, 2008; Shilatifard, 2012; Cenik and Shilatifard, 2021). Furthermore, the COMPASS complex is subject to dynamic regulation, as various signaling pathways and interacting proteins influence its activity. Dysregulation of COMPASS components or its methyltransferase activity can result in abnormal gene expression, highlighting its significance in both development and disease (Cenik and Shilatifard, 2021). Recent whole exome sequencing of schizophrenia cases identified loss-of-function variants in *SETD1A* that are associated with various neurodevelopmental disorders, including schizophrenia (SCZD; OMIM 181500) (Takata et al., 2014; Wang et al., 2022; Nagahama et al., 2020; Singh et al., 2016). Amongst different chromatin modifiers we queried in VarElect database (Stelzer et al., 2016), SETD1A showed a high disease-causing likelihood (Table 3). As such, understanding the role of the COMPASS complex and its regulatory mechanisms offers valuable insights into how H3K4 methylation orchestrates gene expression and contributes to cellular function and neural development.

**Table 1:** Chromatin modifiers causing neurodevelopmental disorders and their speculated mechanisms of pathogenesis.

Enzyme	Function	Enzyme family	Disease	Mechanisms of pathogenesis
Set1A	Di- and trimethylation of H3K4	COMPASS family of histone methyltransferases	Schizophrenia (OMIM 181500)	Haploinsufficiency and loss of function mutations leading to dysregulation of H3K4 methylation.
KMT2D	Trimethylation of H3K4	COMPASS-related family of histone methyltransferases	KABUK1 (OMIM 147920)	Heterozygous mutations are linked to dysregulated H3K4me3 metabolism.
KMT2A	Trimethylation of H3K4	COMPASS-related family of histone methyltransferases	Wiedemann-Steiner syndrome (OMIM 605130)	Dysregulation of H3K4me3 turnover.
SETD2	Trimethylation of H3K36	SET domain-containing family	Luscan-Lumish syndrome (OMIM 616831)	Heterozygous mutations linked to impaired DNA repair and dysregulated transcription.
NSD1	Mono and di-methylation of H3K36	SET domain-containing family	Sotos syndrome 1 (OMIM 117550) ASD	Loss of function of NSD1 linked to dysregulated H3K36me2 deposition. Haploinsufficiency of NSD1.
NSD2	Mono and di-methylation of H3K36	SET domain-containing family	Wolf–Hirschhorn syndrome (OMIM 194190) Rauch–Steindl syndrome (OMIM 619695)	Variable-sized deletions of NSD2. Truncating variants of NSD2 linked to the dysregulated turnover of H3K36me2.
ASH1L	Mono and di-methylation of H3K36	*Trithorax (Trx) family*	Tourette syndrome (TS), autism spectrum disorder (ASD), intellectual disability (IDD), ADHD, and schizophrenia	*ASH1L* mutations been linked to dysregulated turnover of H3K36me2 in multiple NDDs.
EHMT1	Di-methylation of H3K9	SET domain-containing family	Kleefstra syndrome(OMIM 610253) Global developmental delay (GDD)	Mutations or deletions of the EHMT1 been linked to dysregulated turnover of H3K9me2.
SETDB1	Mono, di-, and trimethylation of H3K9	SET domain-containing family	SCZ ASD	Increased expression of SETDB1 and increased H3K9me2 levels have been reported in cortical brain tissue with SCZ. Mutations in SETDB1 been linked to ASD.
EZH2	Tri-methylation of H3K27	SET domain-containing family	Weaver syndrome (OMIM 277590)	Mutations in EZH2 lined to global depletion of H3K27me3.
LSD1	Demethylation of mono- and di-methylated H3K4	Flavin-adenine dinucleotide (FAD)-dependent family of amine oxidases	Kabuki syndrome, KBG syndrome (OMIM 148050)	Missense mutations.
KDM5C	Demethylation of mono- and di-methylated H3K4	alpha-ketoglutarate-dependent hydroxylase	Mental retardation, X-linked, syndromic, Claes-Jensen type (OMIM 300534) Epilepsy	De novo mutations.
KDM2A	Demethylation of di-methylated H3K36	Alpha-ketoglutarate-dependent hydroxylase	IDD	*de novo* mutations.
PHF8	Demethylation of di-methylated H3K9	Alpha-ketoglutarate-dependent hydroxylase	Siderius X-linked mental retardation syndrome (OMIM 300560)	Hemizygous loss-of-function.
KDM6B	Demethylate di- and trimethylated H3K27	Alpha-ketoglutarate-dependent hydroxylase	Stolerman neurodevelopmental syndrome (OMIM 618505)	Heterozygous variants.
DNMT1	Methylation of CG	DNA methyltransferase family	Hereditary sensory and autonomic neuropathy type 1 syndrome (OMIM 614116) Autosomal dominant cerebellar ataxia, deafness, and narcolepsy (OMIM 604121)	Mutations in DNMT1.
DNMT3A	De novo methylation of CH and setting up genomic imprints	DNA methyltransferase family	Tatton-Brown Rahman syndrome (OMIM 615879) ASD	Heterozygous mutations of DNMT3A. Heterozygous mutations of DNMT3A.
DNMT3B	De novo methylation of CH and setting up genomic imprints	DNA methyl transferase family	Immunodeficiency-Centromeric Instability-Facial Anomalies Syndrome 1 (OMIM 242860)	Mutations in DNMT3B.

**Table 2:** Summary of the pathogenic involvement of histone methyltransferases and demethylases, and DNA methylases and demethylases in human neurodevelopmental syndromes.

Epigenetic enzyme	Function	Associated neurodevelopmental syndrome(s)	OMIM number
Histone methyltransferases			
SETD1A	H3K4 methylation	Epilepsy, early onset with or without developmental delay Neurodevelopmental disorder with speech impairment and dysmorphic facies	618,832 619,056
SETD1B	H3K4 methylation	Intellectual developmental disorder with seizures and language delay	619,000
EHMT1 (GLP)	H3K9 methylation	Kleefstra syndrome	610,253
NSD1	H3K36 methylation	Sotos syndrome	117,550
KMT2D (MLL2)	H3K4 methylation	Kabuki syndrome 1	147,920
KMT2A (MLL1)	H3K4 methylation	Wiedemann-Steiner syndrome	605,130
Histone demethylases			
KDM5C (JARID1C)	H3K4 demethylation	Claes-Jensen syndrome	300,534
KDM6A (UTX)	H3K27 demethylation	Kabuki syndrome 2	300,867
KDM6B	H3K27 demethylation	Stolerman neurodevelopmental Syndrome	618,505
DNA methylases (DNMTs)			
DNMT1	Maintenance DNA methylation	Cerebellar ataxia, deafness, and narcolepsy Neuropathy, hereditary sensory, type IE	604,121 614,116
DNMT3A	De novo DNA methylation	Tatton-Brown-Rahman syndrome	615,879
DNMT3B	De novo DNA methylation	Immunodeficiency-centromeric instability-facial anomalies syndrome	242,860
DNA demethylases (TETs)			
TET3	DNA demethylation	Beck-Fahrner syndrome	618,798

Information retrieved from OMIM as of 3/12/2025.

**Table 3:** Disease-causing Likelihood for the genes related to epigenetics in human neurodevelopmental disorders Relevant genes selected form Table 2.

Symbol	Description	GIFTs	Matched phenotypes count	Global rank (Total genes 17,192) (A)	-log(P)	Score (B)	Average disease causing likelihood (C)
KDM5C	Lysine demethylase 5C	59	1	156	2.33	**8.29**	93.05%
DNMT3A	DNA methyltransferase 3 Alpha	65	1	521	1.80	**4.82**	86.72%
DNMT1	DNA methyltransferase 1	66	1	1,031	1.51	**2.52**	73.22%
SETD1A	SET domain containing 1A, Histone lysine methyltransferase	54	1	63	2.72	**10.77**	69.43%
KMT2A	Lysine methyltransferase 2A	61	1	159	2.32	**8.20**	68.59%
EHMT1	Euchromatic histone lysine methyltransferase 1	59	1	95	2.54	**9.45**	68.20%
TET3	Tet methylcytosine dioxygenase 3	53	1	293	2.05	**6.51**	66.52%
DNMT3B	DNA methyltransferase 3 Beta	64	1	906	1.56	**2.88**	63.53%
KDM6A	Lysine demethylase 6A	60	1	830	1.60	**3.13**	61.14%
KMT2D	Lysine methyltransferase 2D	57	1	288	2.06	**6.59**	60.55%
NSD1	Nuclear receptor binding SET domain protein 1	57	1	463	1.85	**5.17**	55.94%
KDM6B	Lysine demethylase 6B	58	1	326	2.01	**6.19**	40.33%
SETD1B	SET domain containing 1B, histone lysine methyltransferase	52	1	283	2.07	**6.63**	0.00%

Analysis performed with VarElect (Stelzer et al., 2016).

(A) Global rank (x relevant genes found): as a first step, the VarElect algorithm searches for all of the genes in the human genome that are related to the queried phenotypes. It then calculates a relevancy “phenotype to gene score” for each one of the genes, and sorts them accordingly. Global rank is basically the rank of a gene in that sorted list, before designating only the genes of interest (those entered by the user and the inferring genes). This value gives another perspective to the results of the analysis by showing how many genes in the whole human genome have any connection to the entered phenotype/s, and where the queried genes rank along that list.

(B) This score is an indication of the strength of the connection between the gene and the queried phenotype/s. Scores usually range from 1 to 200. The main purpose of the score is to enable ranking and prioritizing the list of queried genes by relevance to the queried phenotype/s. Scores from different runs cannot and should not be compared, since relevance scores produced for each run are relative within the run and not absolute. The green bar represents the ratio of each score/maximum score of the current run. When analyzing GeneHancer regulatory elements, the phenotype score is adjusted as follows: Each gene-GeneHancer association has a total score, calculated by multiplying the GeneHancer confidence score by the GeneHancer–Gene association score. Total scores are normalized to a range of 0.05–0.8, and each gene-phenotype score of the regulatory element gene target is multiplied by the normalized GeneHancer total score.

(C) The disease causing likelihood column reflects the principle that a variant in a gene with high mutation intolerance is more likely to be disease causing. RVIS is residual variation intolerance score (Petrovski et al., 2013)and GDI is Gene Damage Index (Itan et al., 2015). Disease- causing likelihoods are 100% minus RVIS percentile or 100% minus GDI percentile (orange bars), with the average of both shown numerically. ND, not determined; NA, not applicable.

##### 1.1.3.3 MLL

The MLL family of proteins, also referred to as KMT2 (lysine methyltransferase 2), represents another crucial group of histone methyltransferases responsible for catalyzing the trimethylation of histone H3 at lysine 4 (H3K4me3) (Sugeedha et al., 2021). This particular histone modification, H3K4me3, is strongly associated with transcriptionally active genome regions, particularly at the promoters of actively transcribed genes. The MLL/KMT2 family plays a fundamental role in maintaining chromatin dynamics and gene expression patterns across various cellular processes, including development, stem cell maintenance, and differentiation (Sugeedha et al., 2021).

The MLL family consists of several members, including MLL1 (KMT2A), MLL2 (KMT2B), MLL3 (KMT2C), MLL4 (KMT2D), and KMT2F (MLL5). These enzymes function as multi-subunit complexes, where the catalytic activity is provided by the SET domain within the MLL proteins located near the *C*-terminus for their intrinsic methyltransferase activity, which is weak in the absence of the core subunits (WDR5, RBBP5, ASH2L, and DPY30) (Ruthenburg et al., 2007). The MLL complexes also recruit various cofactors and regulatory proteins that are essential for the recognition of target genes and the efficient deposition of the H3K4me3 mark (Sugeedha et al., 2021). The interaction of MLL proteins with other chromatin-regulating complexes and transcriptional machinery is crucial for properly activating genes during developmental processes. In the context of development, the MLL family plays a key role in regulating the expression of genes required for cell fate determination and lineage specification. In particular, MLL proteins are essential for stem cell maintenance and differentiation (Artinger et al., 2013; Dai et al., 2017; Goveas et al., 2021). In embryonic stem cells and other progenitor cell types, MLL complexes facilitate the transcription of key pluripotency genes and developmental regulators, helping to maintain a balance between self-renewal and differentiation (Artinger et al., 2013; Milne et al., 2002; Milne et al., 2005; Yu et al., 1995).

The most well-studied neurodevelopmental disorders associated with dysregulated MLL complex are Kabuki syndrome 1 and Wiedemann-Steiner syndrome. Kabuki syndrome 1 is a rare congenital syndrome characterized by a distinctive face, autism, seizure, and microcephaly (KABUK1; OMIM 147920). In more than 50% of patients with KABUK1, heterozygous mutations in *KMT2D* are linked to dysregulated H3K4me3 levels. Similarly, dysregulation of H3K4me3 turnover has been reported in *KMT2A*-deficient cortical neurons of mouse model of Wiedemann-Steiner syndrome. Wiedemann-Steiner syndrome (WDSTS; OMIM 605130), associated with mutations in *KMT2A,* is another extremely rare neurodevelopmental condition accompanied by microcephaly, short stature, autism-like phenotype, and aggression (Strom et al., 2014; Gupta et al., 2010).

#### 1.1.4 H3K36 methyltransferases

H3K36 methyltransferase enzymes are a group of proteins responsible for adding methyl groups to the lysine 36 residue (K36) of histone H3. This methylation, particularly trimethylation (H3K36me3), is crucial in various cellular processes, including transcriptional elongation, alternative splicing, chromatin remodeling, and DNA repair. *In vitro* and *in vivo* studies conducted so far have identified eight types of KMTs that regulate the methylation levels of H3K36 in humans. These include SETD2, SETD3, NSD1, NSD2, NSD3, ASH1L, SMYD2, and SETMAR. These enzymes belong to the SET domain-containing family of methyltransferases, characterized by a conserved SET [Su(var)3–9, Enhancer-of-zeste, Trithorax] domain responsible for the methylation activity (Hyun et al., 2017).

Beyond its involvement in transcriptional elongation, H3K36me3 also plays an essential role in maintaining transcriptional fidelity by preventing inappropriate transcriptional initiation and suppressing spurious transcriptional activity, through interaction with DNMT3A and DNA methylation (Dhayalan et al., 2010; Hyun et al., 2017). Specifically, H3K36me3 helps inhibit transcription initiation in the gene body, preventing the reactivation of previously silenced genes and ensuring that transcription is restricted to the correct regulatory regions. By acting as a barrier to unregulated transcriptional initiation, H3K36me3 helps protect against the aberrant activation of genes that could lead to cellular dysfunction (Sessa et al., 2019; Wagner and Carpenter, 2012). The regulation of H3K36 methylation is particularly important in NDD, as alterations in H3K36 methylation have been linked to various neurological conditions (Hamagami et al., 2023; Kinoshita et al., 2024). For example, mutations in the SETD2 gene, which encodes a methyltransferase responsible for H3K36me3, have been associated with ASD (case report) and neurodevelopmental disorders with or without macrocephaly overgrowth, highlighting the importance of this modification in brain development (Chen et al., 2021; Pappas and Rabin, 1993). SETD2 mutations reduce H3K36me3 levels, resulting in defects in transcriptional elongation and causing dysregulation of gene expression that can impact neuronal development and function (Zaghi et al., 2019).

In addition, H3K36me3 has been implicated in regulating alternative splicing, a critical process for neuronal diversity (Jia et al., 2024; Xu et al., 2021; Hu et al., 2017). Aberrant H3K36 methylation can disrupt the splicing of pre-mRNA, producing nonfunctional or malfunctioning proteins that can impair neuronal function and contribute to cognitive deficits. Studies have shown that neurodegenerative diseases, including ASD and intellectual disabilities, often involve disturbances in the regulation of H3K36 methylation, further emphasizing its significance in neurodevelopment and neuronal homeostasis (Zaghi et al., 2019). Moreover, cancer-related neurodevelopmental disorders may also result from disruptions in H3K36 methylation. Alterations in the transcriptional regulation of genes linked to neural differentiation, growth, and survival contribute to abnormal brain development (Zaghi et al., 2019). The connection between H3K36 methylation and these conditions highlights the critical role of precise transcriptional regulation in the developing brain.

In summary, H3K36me3 is a vital epigenetic mark that plays multiple roles in maintaining proper transcriptional regulation during gene expression. Beyond its critical function in transcriptional elongation, it ensures the fidelity of gene expression by preventing inappropriate transcriptional initiation and protecting the integrity of the transcriptome. Disruptions in regulating H3K36 methylation have profound implications for neurodevelopment. They are implicated in a range of neurodevelopmental disorders, further underscoring the importance of this modification in proper brain function and development.

#### 1.1.5 SETD2 (SET domain containing 2)

SETD2 is the most well-characterized histone H3K36 methyltransferase, known for its critical role in catalyzing the addition of three methyl groups to histone H3 at lysine 36 (H3K36me3) (Molenaar and van Leeuwen, 2022). This specific histone modification is a hallmark of actively transcribed gene bodies and is essential for transcriptional elongation, ensuring the proper progression of RNA polymerase during gene expression. However, SETD2’s role extends far beyond transcriptional regulation; it is integral to maintaining genomic stability and coordinating essential DNA repair mechanisms (Molenaar and van Leeuwen, 2022). In cellular physiology, SETD2 plays a significant role in genome maintenance, particularly in processes related to mismatch repair (MMR) and double-stranded DNA break repair (DSBR) (Molenaar and van Leeuwen, 2022; McDaniel and Strahl, 2017). By catalyzing H3K36me3, SETD2 facilitates the recruitment of various DNA repair factors to sites of DNA damage, ensuring the fidelity of DNA replication and repair (Carvalho et al., 2014; Li et al., 2013; Molenaar and van Leeuwen, 2022; McDaniel and Strahl, 2017).

Beyond its role in DNA repair, SETD2 profoundly impacts the nervous system, particularly in the context of neurodegenerative diseases (Pappas and Rabin, 1993). The first notable interaction of SETD2 within the nervous system was its discovery in association with the Huntington’s disease (HD) protein (Faber et al., 1998; Seervai et al., 2020). HD, a neurodegenerative disorder characterized by progressive motor dysfunction, cognitive decline, and psychiatric symptoms, is caused by mutations in the *HTT* gene, which encodes the Huntingtin protein (HTT) (Faber et al., 1998). SETD2 was found to interact directly with the mutated form of the Huntington protein, and this interaction is thought to contribute to the disease’s pathology. Specifically, SETD2’s involvement in HD appears to disrupt normal gene expression regulation, impair DNA repair processes, and exacerbate the neurodegenerative effects of the disease (Edmunds et al., 2008; Faber et al., 1998; Seervai et al., 2020). The dysregulation of SETD2-mediated H3K36 methylation, mainly through its failure to effectively regulate transcription and repair DNA damage, can lead to neuronal dysfunction and contribute to neurodegenerative processes (Pappas and Rabin, 1993; Lumish et al., 2015). Moreover, the loss of SETD2 activity, which results in reduced H3K36me3, can impair transcriptional elongation, causing dysregulation of critical genes involved in neuronal survival, synaptic plasticity, and neurogenesis (Pappas and Rabin, 1993). This disruption may lead to the accumulation of defective proteins, increased oxidative stress, and the development of neurodegenerative symptoms.

SETD2 mutations and dysregulation have also been implicated in other NDD, particularly in compromised genome stability. Recently, *de novo* SETD2 variants have been reported in neurodevelopmental disorders including autism spectrum disorder (ASD). For example, heterozygous mutations in SETD2 have been linked to Luscan-Lumish syndrome (LLS; OMIM 616831), characterized by macrocephaly, postnatal overgrowth, ASD, developmental delay (D'Gama et al., 2015; Lumish et al., 2015; Wu et al., 2023). For instance, SETD2-related disorders often exhibit intellectual disabilities, developmental delays, and heightened susceptibility to cancer as a result of impaired DNA repair and transcriptional regulation (Pappas and Rabin, 1993). These findings suggest that SETD2 is not only a key player in maintaining genomic integrity but also plays a critical role in ensuring the proper development and function of the nervous system.

In summary, SETD2’s interaction with the HTT (Faber et al., 1998) in the nervous system exemplifies its critical role in neurodegenerative disease pathology. The dysregulation of SETD2 and H3K36 methylation has profound implications for neurodevelopment, contributing to neurodegenerative diseases, intellectual disabilities, and other related disorders. Understanding SETD2’s function in both transcriptional regulation and DNA repair processes offers valuable insights into its role in maintaining neuronal health. It offers potential avenues for therapeutic interventions in neurodegenerative and neurodevelopmental diseases.

#### 1.1.6 NSD family (nuclear SET domain-containing proteins)

The NSD family of proteins (e.g., NSD1, NSD2, NSD3) function in broader chromatin remodeling activities. These enzymes are primarily linked to H3K4me2, H3K4me3, and H3K36 methylation. NSD family members are implicated in gene regulation during development and differentiation and are often associated with oncogenesis when dysregulated. NSD1 is the methyltransferase responsible for mono and di-methylation of lysine 36 of histone H3 (Hamagami et al., 2023). Interestingly, *Nsd1* conditional knockout mice display defects in spatial memory, motor learning, and coordination, likely due to dysregulation of cortico-thalamus-cortical wiring. Mechanistically, H3K36me2 deposition mediated by NSD1 seems to be required to establish and maintain region- and layer-specific neocortical identities (Zheng et al., 2023). In humans, loss of function of NSD1 gene has been linked to Sotos syndrome 1 (SOTOS1; OMIM 117550) (Kurotaki et al., 2002; Saugier-Veber et al., 2007), whereas haploinsufficiency of NSD1 has been linked ASD (Satterstrom et al., 2020). SOTOS1 is an autosomal dominant condition characterized by pre- and postnatal overgrowth, distinctive facial features, macrocephaly, and non-progressive developmental delay (Kurotaki et al., 2002). Interestingly, duplication of NSD1 results in microcephaly in 74% of 5q35 syndrome cases, along with growth restriction and developmental delay in nearly 90% of affected individuals (Quintero-Rivera et al., 2021).

### 1.2 NSD2

NSD2 (MMSET or WHSC1) mediates histone H3 lysine 36 mono- and di-methylation (H3K36me2) (Kuo et al., 2011). Variable-sized deletions in chromosome 4p16.3, including NSD2, cause Wolf–Hirschhorn syndrome (also known as 4p-syndrome; WHS; OMIM 194190), which is characterized by growth retardation, developmental delay/intellectual disability (DD/ID), microcephaly, hypotonia, and congenital malformations (Bergemann et al., 2005). Recent human genetic studies have discovered another ID linked to missense and truncating variants of NSD2 called Rauch–Steindl syndrome (RAUST; OMIM 619695) (Zanoni et al., 2021). RAUST phenotype represents a mild form of Wolf-Hirschhorn syndrome. Although we have extensive knowledge of NSD2’s role in hematopoiesis and oncogenesis, its function in brain development remains poorly understood. In a mouse model of WHS with deletions of the *Nsd2* gene, the animals exhibited growth retardation, midline, craniofacial, and ocular abnormalities, but showed no signs of learning deficits (Naf et al., 2001). A more recent transcriptome study on the E15.5 brains of control and *Nsd2* KO mice revealed that the loss of NSD2 caused dysregulation of genes related to synaptic transmission and formation (Kinoshita et al., 2024). Intriguingly, despite the global loss of H3K36me2 in *Nsd2* KO brains, the loss of NSD2 did not wholly deplete H3K36me2, suggesting that NSD1 and NSD2 redundantly contribute to the broad H3K36me2 pattern in the nervous system (Kinoshita et al., 2024).

### 1.3 ASH1L

ASH1L (Absent, Small, or Homeotic Discs-1-Like) is a histone methyltransferase that plays a crucial role in neurodevelopment by regulating gene expression through catalyzing the mono- and di-methylation of H3K36 (Gregory et al., 2007). These modifications are generally associated with transcriptional activation, gene elongation during transcription, and chromatin remodeling. ASH1L is highly expressed in the brain during development and occupies the transcribed region of active genes, like *Hox* genes. Not surprisingly, ASH1L mutations have been associated with the neuropathology of Tourette syndrome (TS), autism spectrum disorder (ASD), intellectual disability (IDD), Attention Deficient Hyperactivity Disorder (ADHD), and schizophrenia (Zhang et al., 2021; Gao et al., 2021; Liu et al., 2020). In addition to ASD and ID, patients with *ASH1L* mutations also display a variety of developmental and behavioral abnormalities, including delayed myelination, microcephaly, craniofacial deformity, skeletal abnormality, and feeding difficulties, suggesting critical roles of *ASH1L* in normal embryonic and postnatal development (Shen et al., 2019; Okamoto et al., 2017; de Ligt et al., 2012). Interestingly, the mouse model carrying loss of *Ash1L* manifested autistic-like behavior (Gao et al., 2021). Mechanistically, multiple genes critical for normal brain development and highly related to human ASD were found to have reduced expression in the differentiating *Ash1L-*knock out NPCs (Gao et al., 2021). This suggests that ASH1L might function as a master epigenetic regulator to facilitate the expression of genes (such as *FOXG1*) critical for normal brain development.

Consistent with above reports, Lalli et al. showed that inhibition of *ADNP, ASH1L, CHD2,* and *DYRK1A* delays neuronal differentiation through a shared transcriptional pathway involved in cell cycle control and neural progenitor cell proliferation (Lalli et al., 2020). In addition to regulating neural progenitors cell cycle and differentiation, ASH1L also monitors neuronal morphogenesis by modulating neurotrophin signaling, especially the BDNF–TrkB pathway, to affect neuronal arborization (Cheon et al., 2022). Consistently, mutation in ASH1L lowers the expression of TrkB in the brain, preventing neurons from responding to exogenous BDNF, and leading to reduced neurite outgrowth (Wong et al., 2015).

In a neuropathological context, patients with seemingly disparate disorders like TS, ASD, ADHD, and SCZ exhibit common abnormalities, including long-distance “underconnectivity” and short-range “overconnectivity,” which suggests a shared final pathway (van den Heuvel and Sporns, 2019; Taniguchi and Moore, 2014). This puts ASH1L at the heart of common networks of co-substrates, which are virtually linked across multiple neuropsychiatric disorders. Future challenges include understanding the complexity of ASH1L interactions and its non-histone substrates (An et al., 2011; Davidovich and Zhang, 2021) and determining the extent to which cognitive deficits result from the loss of ASH1L activity, either in the formation of circuits or in maintaining their homeostasis and function.

#### 1.3.1 H3K9 methyltransferases

Several enzymes are involved in the methylation of histone H3 at lysine 9 (H3K9), a modification that is crucial for the regulation of various biological processes, including gene silencing, heterochromatin formation, and the maintenance of genomic stability. H3K9 methylation exists in different forms, with H3K9me1, H3K9me2, and H3K9me3 representing mono-, di-, and tri-methylation of H3K9, respectively. Of these, H3K9me2 and H3K9me3 are particularly important and are strongly associated with the formation of heterochromatin, a highly condensed chromatin structure that is transcriptionally silent (Padeken et al., 2022). The addition of methyl groups to H3K9 is catalyzed by a variety of histone methyltransferases (HMTs), including SUV39H1, SUV39H2, and EHMT1/2, which play a central role in establishing and maintaining these repressive chromatin states (Padeken et al., 2022). The H3K9me2 and H3K9me3 modifications are recognized by specific chromatin-binding proteins, such as the HP1 (heterochromatin protein 1) family, which recruits additional factors that further promote chromatin compaction and gene silencing (de Ligt et al., 2012; Meyer-Nava et al., 2020). These modifications are key to maintaining transcriptional repression in genome regions that must remain inactive, such as centromeres, telomeres, and transposon regions (Padeken et al., 2022; Poleshko et al., 2019). Heterochromatin, marked by these methylation states, is a tightly packed chromatin configuration that ensures that unnecessary or potentially harmful gene expression does not occur. This repression is vital for preserving genome integrity, as activating transposons or repetitive elements can lead to genomic instability and disrupt cellular function (Padeken et al., 2022).

In addition to heterochromatin formation, H3K9 methylation plays a key role in X-chromosome inactivation in female mammals (Keniry et al., 2016; Silva et al., 2003; Boggs et al., 2002). This process ensures that one of the two X chromosomes in each female cell is transcriptionally silenced to balance gene dosage between males (one X chromosome) and females (two X chromosomes). The H3K9me3 mark is deposited on the inactive X chromosome, contributing to its stable silencing and preventing the expression of the genes on that chromosome (Keniry et al., 2016).

The disruption of these H3K9 methyltransferases has been implicated in a variety of NDDs, leading to abnormal gene expression, impaired neuronal development, and cognitive deficits (Ciptasari and van Bokhoven, 2020). Dysregulation of these enzymes and their associated modifications has been linked to a wide array of neurodevelopmental disorders, including Rett syndrome, Fragile X syndrome, Huntington’s disease, Angelman syndrome, Prader-Willi syndrome, and other cognitive and developmental disorders.

#### 1.3.2 EHMT1

EHMT1 (also known as G9a-like protein (GLP)) is a histone methyltransferase that plays a key role in neurodevelopment by adding mono- and di-methyl groups to histone H3 at lysine 9 (H3K9me1/2), a modification associated with gene silencing and the regulation of chromatin structure (Padeken et al., 2022). EHMT1’s primary function is maintaining repressive heterochromatin states and regulating gene expression during critical processes like neurogenesis, synaptic plasticity, and neuronal differentiation (Martens et al., 2016). Hippocampal CA1 neurons of *Ehmt1*^+/−^ mice show reduced dendritic branching and spine density (Balemans et al., 2013). Besides, these mice show increased paired-pulse facilitation at the CA3-CA1 synapse, indicative of a presynaptic deficit associated with decreased release probability (Balemans et al., 2013). Consistent with this, new work from Dr. Kasri’s team found that EHMT1 deficiency impaired spontaneous network activity and lowered firing rates during early development. In contrast, basal, action potential-independent excitatory synaptic transmission was unaffected (Martens et al., 2016). In human iPSC-derived neurons, EHMT1 deficiency leads to upregulation of NRSF/REST target genes, which results in elevated expression of the human pro-neural transcription factors MASH1 and NGN2 (Alsaqati et al., 2022).

One of the most well-known disorders associated with EHMT1 mutations is Kleefstra syndrome (KS; OMIM 610253), a rare genetic condition characterized by intellectual disability (ID), childhood hypotonia, and distinctive facial features (Kleefstra et al., 2006; Kleefstra et al., 2005; Kleefstra et al., 2012). In Kleefstra syndrome, mutations or deletions of the EHMT1 gene impair its ability to regulate chromatin structure, leading to abnormal gene expression that disrupts neuronal development and function. This disruption is thought to affect the normal processes of synaptic plasticity, cognitive development, and motor coordination, contributing to the characteristic symptoms of the syndrome. A recent study suggested that the developmental impairments observed in EHMT1-deficient networks may result in a temporal misalignment between activity-dependent developmental processes, thereby contributing to the pathophysiology of Kleefstra syndrome (Martens et al., 2016). Intellectual disability (IDD) in Kleefstra syndrome typically ranges from mild to moderate, and individuals often experience delays in speech and language development (Balemans et al., 2013; de Boer et al., 2018). Childhood hypotonia, or low muscle tone, is another hallmark feature that affects motor skills and leads to developmental delays in milestones such as sitting, crawling, and walking. In addition to these developmental challenges, Kleefstra syndrome is distinguished by a unique facial phenotype, which may include features like a broad forehead, deep-set eyes, wide nasal bridge, and a thin upper lip. Other common clinical features include heart defects, seizures, and behavioral issues, such as ASD or repetitive behaviors behaviors (Deimling et al., 2017; de Boer et al., 2018; Ausio et al., 2003).

The role of EHMT1 in Kleefstra syndrome highlights the significance of proper H3K9 methylation in regulating gene expression during neurodevelopment. Since EHMT1 catalyzes the H3K9me2 modification, a crucial mark linked to gene silencing, mutations in this enzyme can result in the inappropriate activation or silencing of genes essential for neuronal differentiation, function, and maintenance. This dysregulation contributes to the complex phenotype observed in Kleefstra syndrome. Beyond Kleefstra syndrome, mutations in EHMT1 have been implicated in other neurodevelopmental disorders, including ASD and global developmental delay (GDD) (Kleefstra et al., 2012). These findings highlight the critical role of H3K9 methylation in regulating brain development and function. They emphasize the need for further research into EHMT1 and its role in chromatin regulation, as well as potential therapeutic approaches that could target this pathway to mitigate the impact of EHMT1 mutations on neurodevelopment.

#### 1.3.3 SETDB1

Among all these H3K9 HMTs, SETDB1 is the only one that catalyzes all three forms of methylation (mono-, di-, and tri-) *in vivo* and forms various repressive protein complexes both at euchromatic and heterochromatic regions (Padeken et al., 2022). Through binding with KAP-1 or coordinating with the DNA methylation machinery, SETDB1 can target multiple genomic loci in different cell types or at different stages during brain development (Padeken et al., 2022).In the early stages of brain development, Setdb1 is highly expressed in proliferating neural progenitor cells. However, its expression gradually decreases towards the later stages of brain development when transition from neurogenesis to differentiation occurs (Figure 2) (Tan et al., 2012). Consistent with this, dysregulation or mutations in SETDB1 have been linked to various neurological disorders, such as SCZ and ASD. Multiple lines of evidence support the role of SETDB1 in the etiology of SCZ. For instance, increased expression of SETDB1 and increased H3K9me2 levels have been reported in postmortem parietal cortical brain tissue with SCZ compared to healthy controls (Gavin et al., 2009; Chase et al., 2013). While the misexpression of SETDB1 has been associated with SCZ, mutations in SETDB1 have also been linked to ASD. In a recent study, Cukier and colleagues discovered an ASD-specific nonsynonymous mutation, Pro1067del, which falls into the catalytic SET domain of SETDB1 protein and is inherited maternally. The incidence of a second variation, Pro529Leu, was significantly increased in patients with ASD compared with controls and was inherited both maternally and paternally (Cukier et al., 2012).

#### 1.3.4 H3K27 methyltransferases

Polycomb Repressive Complex (PRC) is responsible for methylation of H3K27. The core PRC2 complex consists of EZH1/2, EED164, SUZ12, and RBBP4/7, and can associate with several PRC2 accessory proteins, such as PCL1-PCL3, JARID2, AEBP2, EPOP, and LCOR (Margueron et al., 2009; Hansen et al., 2008). PRC1 is made up of RING1A/B and PCGF-PCGF6. This core PRC1 interacts with CBX-family proteins, PHC1-PHC3, and SCMH1/2 to form the canonical PRC1 complex. In contrast, the non-canonical PRC1 contains RYBP/YAF2, KDM2B, DCAF7, and WDR5. It is believed that these two complexes function as an H3K27me3 methyltransferase and as mediators of H2AK119 mono-ubiquitination, respectively (Margueron et al., 2009; Hansen et al., 2008).

Polycomb Repressive Complexes (PRC1 and PRC2) are essential to neurodevelopment, as they regulate the chromatin landscape and ensure the appropriate repression of genes during critical stages of neural differentiation, migration, and maturation (Zhang et al., 2015; Cheon et al., 2022; Roman-Trufero et al., 2009). Their role in maintaining the balance between stem cell self-renewal and differentiation and in neuronal plasticity makes them essential for the proper development of the nervous system (Hansen et al., 2008; Zhang et al., 2015; Lee et al., 2006). Consistent with this, lacking RING1B, a component of PRC1 complex, results in skewed differentiation patterns in mouse embryonic NSCs partially due to miss-regulation of Notch signaling pathway (Roman-Trufero et al., 2009). Suggesting that PRC1 could be involved in the maintenance of the undifferentiated state of embryonic NSCs as well as the differentiation potential of NSCs to neurons and glia. Not surprisingly, dysregulation of PRC components can lead to various neurodevelopmental disorders, underscoring their significance in brain development and function (Ritchie and Lizarraga, 2023). For instance, mutations in components of PRC2 (like EZH2, the catalytic subunit of PRC2) are linked to an array of neurodevelopmental and neuropsychiatric disorders, including IDD, ASD, and SCZ (Tatton-Brown et al., 2013; Cohen et al., 2016; Gao et al., 2024; Gibson et al., 2012; Ritchie and Lizarraga, 2023; Deevy and Bracken, 2019). The effects of PRC dysregulation are particularly severe during embryonic and postnatal brain development, where the precise timing and location of gene expression are crucial for appropriate neuronal wiring, synapse formation, and learning processes (Deevy and Bracken, 2019). PRC dysfunction can result in epigenetic instability, leading to the repression of neurogenesis or inappropriate activation of developmental genes, both of which can drastically affect cognitive abilities and behavior (Cheon et al., 2022; Deevy and Bracken, 2019).

#### 1.3.5 EZH2

Enhancer of zeste homolog 2 (EZH2) catalyzes tri-methylation on histone 3 lysine 27 (H3K27me3), modification that represses gene expression, and is highly expressed in early gestation in neural precursor cells where it inhibits differentiation into neurons by blocking Wnt-signaling mediated activation of neural genes (Pereira et al., 2010; Aranda et al., 2015; Zaidi et al., 2011; Cohen et al., 2016; Tatton-Brown et al., 2013). At differentiation stage of brain development, EZH2 is required for switching Intermediate Progenitors cells fate from neurogenic to astrogenic by H3K27 trimethylation of Neurog1 promoter (Hirabayashi et al., 2009) (Figure 2).One of the well-known pieces of evidence showcasing the role of PRC2 complex in neurodevelopment comes from Weaver syndrome (WVS) studies. Caused by mutations in EZH2 (WVS; OMIM 277590), Weaver’s syndrome is an autosomal dominant disease characterized by learning disabilities, dysmorphic facial features and general overgrowth, which can include tall stature, obesity and macrocephaly (Gibson et al., 2012). Recently, a mouse model for the most common Weaver syndrome missense variant, *EZH2* p.R684C was developed, and mouse embryonic fibroblasts (MEFs) obtained from these animals showed global depletion of H3K27me3. In addition, these mice had abnormal bone parameters indicative of skeletal overgrowth reminiscent of WVS (Gao et al., 2024). Future research is needed to explore the locus-specific role of H3K27 methylation in growth regulation and brain size development, which could provide insight into the molecular mechanisms underlying Weaver syndrome and other related PRC2 syndromes.

#### 1.3.6 Histone demethylases

Two distinct classes of enzymes exhibiting histone lysine demethylase activity have been identified to date. LSD1/KDM1 exclusively represents the inaugural class, whereas the remaining known demethylases are categorized within the jumonji (JmjC)-domain-containing class (Nottke et al., 2009). The first class follows an amine oxidase-domain-containing mechanism using FAD as a co-factor to catalyze an amine oxidation of the protonated nitrogen. This reaction releases a formaldehyde molecule per reaction, resulting in a de-methylated lysine. This de-methylated lysine can also undergo the same reaction to become unmethylated. The JmjC-domain-containing mechanism, on the other hand, uses co-factors Fe(II), O_2,_ and *α*-ketoglutarate to hydroxylate the methyl group. The reaction then proceeds analogously to an LSD1/KDM1 demethylation, with the unstable carbinolamine group being spontaneously released as formaldehyde (Nottke et al., 2009).

Currently, six families of KDMs have been described, namely KDM1 to KDM6. Following this, we will discuss the members of each family and their dysregulation in NDDs (Tables 1, 2).

#### 1.3.7 H3K4 demethylases

KDM1A and KDM5C (LSD1) catalyze the demethylation of mono- and di-methylated H3K4, whereas KDM5A catalyzes the demethylation of tri-methylated H3K4 (Nottke et al., 2009). In early brain development, KDM5C is highly expressed in the VZ and SVZ, which expression is required for downregulation of cell-cycle inhibitor p21 and upregulation of Atrophin1 (ATN1) (Zhang et al., 2014). A missense mutation in the amine oxidase domain of KDM1A has been reported in patients exhibiting mixed features of Kabuki syndrome (KABUK1) and KBG syndrome (KBGS; OMIM 148050), both of which are characterized by macrodontia, distinct craniofacial features, and IDD (Tunovic et al., 2014). Similarly, mutations in KDM5C (also known as JARID1C) have been linked to intellectual disability, X-linked, syndromic, Claes-Jensen type (MRXSCJ; OMIM 300534), and epilepsy (Najmabadi et al., 2011; Jensen et al., 2005; Tahiliani et al., 2007; Poeta et al., 2013; Lintas et al., 2022; Peng et al., 2015; Goncalves et al., 2014). Intriguingly, amongst different chromatin modifiers we queried in VarElect database (Stelzer et al., 2016), KDM5C showed the highest disease-causing likelihood (Table 3). This reflects the principle that a variant in KDM5C is highly likely to cause human NDD.

#### 1.3.8 H3K36 demethylases

KDM2A and KDM4B are known to demethylate H3K36me2 and H3K36me3 (Nottke et al., 2009). KDM2A.

Selectively removes mono- and dimethyl groups from histone H3K36, whereas KDM4B has a broader range of substrates by demethylating H3K9me3/me2, H3K36me3/me2, H1.4K26me3, and H4K20me3 (Wilson and Krieg, 2019). KDM4A removes di- and tri-methyl groups from H3K9me2/3, H3K36me2/3, and H1.4K26me2/me3, regulating gene expression (Yang et al., 2024; Whetstine et al., 2006). Recent research identified *de novo* mutations at the *KDM2A* locus in individuals with varying degrees of IDD. These mutations significantly decreased the expression of the KDM2A protein, which appears to impair the proliferation of neural progenitor cells (NPCs), increase apoptosis, induce premature neuronal differentiation, and affect synapse maturation (Ren et al., 2024).

#### 1.3.9 H3K9 demethylases

H3K9me2 is a substrate for KDM4A and PHF8. Interestingly, PHF8 is a dual-function protein; that is the PHD domain preferentially recognizes trimethylated H3K4 (H3K4me3), whereas the catalytic JmjC domain is responsible for preferential demethylation of H3K9me2 (Horton et al., 2010; Feng et al., 2010; Yu et al., 2010). Thus, PHF8 positively regulates gene expression, dependent on its H3K4me3-binding PHD and catalytic domains. H3K9me2 is an evolutionarily conserved, tissue-specific mark and is retained through mitosis (Poleshko et al., 2019). The significance of H3K9me2 may be highlighted during the early stages of brain development by monitoring the nuclear physiology of neural stem and progenitor cells (Qi et al., 2010). Consistent with the above, hemizygous loss-of-function variants in PHF8 have been associated with Siderius X-linked Mental Retardation Syndrome (MRXSSD; OMIM 300560), characterized by features such as hypertelorism, microcephaly, an elongated face, ptosis, and mild facial asymmetry (Siderius et al., 1999; Laumonnier et al., 2005; Koivisto et al., 2007; Ibarluzea et al., 2020; Sobering et al., 2022).

#### 1.3.10 H3K27 demethylases

KDM6A and KDM6B demethylate di- and trimethylated H3K27 through the catalytic activity of the iron-containing jumonji C (JmJC) domain (Jones et al., 2018; De Santa et al., 2007). Pathogenic mutations are linked to Kabuki syndrome 2 (OMOM 300867). Kabuki syndrome is a congenital neurodevelopmental disorder characterized by intellectual disability, postnatal growth deficiency, and distinct facial features, including long palpebral fissures, a broad nasal tip, and prominent earlobes. Additional features may include skeletal abnormalities, persistent fetal fingerpads, and recurrent otitis media in infancy. The syndrome is named for resembling the makeup used in Kabuki theater, a traditional Japanese art form.

According to OMIM, heterozygous variants in *KDM6B* cause “neurodevelopmental disorder with coarse facies and mild distal skeletal abnormalities.” Heterozygous mutation in the KDM6B gene has been linked to Stolerman neurodevelopmental syndrome (NEDSST; OMIM 618505). NEDSST is characterized by developmental delay, often with motor and speech delay, mildly impaired intellectual development (in most patients), learning difficulties, and behavioral abnormalities, including ASD (Stolerman et al., 2019; Rots et al., 2023).

#### 1.3.11 DNA methylases

In the mammalian genome, DNA methylation is an epigenetic mechanism involving the transfer of a methyl group onto the C5 position of the cytosine to form 5-methylcytosine (mC) by the DNA methyltransferase (DNMT). The discovery of the key methyl-DNA–binding protein, methyl-CpG-binding protein 2 (MeCP2), provided evidence of DNA methylation’s critical role in the brain (Chahrour and Zoghbi, 2007). Recent studies from multiple laboratories have revealed widespread cytosine modifications in the brain beyond mCG. These so-called non-CG methylations (mCH, where H = A, C, or T) are now recognized to accumulate in the human and mouse brain after birth, reaching levels comparable to those of mCG in the neuronal genome (Guo et al., 2014). Functionally, methylated CGs (mCGs) and mCHs can inhibit transcription *in vitro* and are recognized by MeCP2 in neurons *in vivo* (Kinde et al., 2016). Intriguingly, loss of MeCP2 leads to the severe X-linked neurological disorder Rett syndrome (RTT), one of the most common causes of neurological impairment in girls (Amir et al., 1999). Duplication of MeCP2, on the other hand, causes MeCP2 duplication syndrome, an ASD, indicating that mCA readout is crucial for brain function (Lyst and Bird, 2015; Van Esch et al., 2005).

#### 1.3.12 DNMT1

Canonical DNMT enzymes include DNMT1, DNMT3A and DNMT3B (Lyko, 2018). Murine *DNA methyltransferase 1* (*Dnmt1*), was the first mammalian DNA methyltransferase cloned by Bestor et al. (1988). DNMT1 preserves the patterns of methylated CG in the mammalian genome during DNA replication and cell division, and it is considered the maintenance DNMT (Yoder et al., 1996). Knocking out *Dnmt1* in mice causes embryonic lethality between E8.0 and E10.5. During this period, the knockout embryos show a two-thirds reduction in DNA methylation and widespread apoptosis in various developing tissues, including the brain (Li et al., 1992). In humans, mutations in *DNMT1* have been identified in hereditary sensory and autonomic neuropathy type 1 (HSAN1) syndrome (HSN1E; OMIM 614116), with other neuropathies and autosomal dominant cerebellar ataxia, deafness, and narcolepsy (ADCADN; OMIM 604121) (Klein et al., 2011; Klein et al., 2013). HSAN1 is extremely rare neurodegenerative disease caused by mutations that lead to misfolding, impaired nuclear localization, and early degradation of DNMT1. However, mutations do not affect the targeting of DNMT1 to the replication foci during cellular replication, but the DNMT1 association with heterochromatin beyond S phase is disrupted. This might be the reason behind 8% reduction in global DNA methylation level, which astonishingly cause neurodegeneration (Moore et al., 2013).

#### 1.3.13 DNMT3A

Unlike most CG methylation, CH methylation is established *de novo* during neuronal maturation and relies on DNA methyltransferase 3A (DNMT3A) for its active maintenance in postmitotic neurons (Xie et al., 2012). Using Dnmt3a heterozygous deletion mice, Dr. Gabel’s team assessed the effect of shared loss-of-function effects resulting from missense and deletion mutations, revealed a global reduction of mCA throughout the brains of Dnmt3a mutants. Besides, loss of mCA leads to disruption of distal regulatory enhancer activity and changes in gene expression that overlap with models of MeCP2 disorders and other NDDs (Christian et al., 2020). In humans, exome sequencing studies have identified heterozygous mutations in DNMT3A in ASD (Satterstrom et al., 2020; Sanders et al., 2015; Iossifov et al., 2014; Feliciano et al., 2019). Besides, specific heterozygous mutations of DNMT3A has been linked to DNMT3A overgrowth syndrome or Tatton-Brown Rahman syndrome (TBRS; OMIM 615879), an NDD characterized by IDD, overgrowth, craniofacial abnormalities, anxiety, and ASD (Tatton-Brown et al., 2014).

#### 1.3.14 DNMT3B

While DNMT3A and DNMT3B are generally recruited to CG-dense regions, DNMT3B is recruited explicitly to the bodies of actively transcribed genes through its PWWP domain interacting with H3K36me3 (Baubec et al., 2015). Consistent with this observation, Neri *et al*. uncovered a significant increase in spurious transcripts originating from cryptic intragenic promoters in *Dnmt3b* KO (Neri et al., 2017) and suggesting that binding of DNMT3B to actively transcribing gene body is crucial for enhancing transcriptional fidelity. This could be of special importance for long neural genes, for which regulation might need several layers of monitoring to safeguard transcript quality. In humans, mutations in DNMT3B have been linked to Immunodeficiency-Centromeric Instability-Facial Anomalies Syndrome 1 (ICF1; OMIM 242860). ICF syndrome is a rare autosomal recessive disease characterized by facial dysmorphism, immunoglobulin deficiency, IDD, recurrent and prolonged respiratory infections, and infections of the skin and digestive system (Valkova et al., 1987; Hagleitner et al., 2008).

The limited expression of Dnmt3b to neural progenitor cells and the mild cognitive defects seen in ICF patients suggest that DNMT3B may be crucial for early neurogenesis. The knockdown of DNMT3B in human embryonic stem cells (hESCs), revealed accelerated maturation with earlier expression of mature neuronal markers (such as *NEUROD1*) and of early neuronal regional specifiers (such as those for the neural crest) (Martins-Taylor et al., 2012). This was accompanied by hypomethylation along the X chromosome and pericentromeric regions, suggesting changes in large-scale methylation patterns due to DNMT3B deficiency alter the timing of neuronal differentiation and maturation (Martins-Taylor et al., 2012).

#### 1.3.15 DNA demethylases

In 2009, the TET (Ten-Eleven Translocation) proteins were identified as enzymes responsible for converting 5-methylcytosine (5mC) into 5-hydroxymethylcytosine (5hmC). This discovery provided a breakthrough in understanding the dynamics of DNA methylation and its role in gene regulation, cellular processes, and epigenetics (Tahiliani et al., 2009). These enzymes, primarily TET1, TET2, and TET3, are involved in the regulation of DNA methylation and hydroxymethylation, which are key to controlling gene expression during brain development (Pastor et al., 2013; Beck et al., 2020; Cochran et al., 2020). Beck-Fahrner syndrome (BEFAHRS; OMIM 618798), caused by homozygous, compound heterozygous, or heterozygous mutation in the *TET3* gene, is a neurodevelopmental disorder characterized by global developmental delay and variable intellectual disability. Individuals often exhibit behavioral differences, including autistic traits, ADHD, and learning disabilities. Common features include hypotonia, distinctive facial characteristics, and growth abnormalities, which may manifest as overgrowth, poor growth, or feeding difficulties. In some cases, seizures may also occur.

The importance of TET enzymes in neurodevelopmental disorders was highlighted by discovery of the marked upregulation of TET1 expression in ASD patients (Zhubi et al., 2014). A more recent study used the genomic DNA isolated from the postmortem cerebellum of both ASD patients and age-matched controls and profiled genome-wide distribution of 5hmC. This study identified age-dependent differentially hydroxymethylated regions (DhMRs) that are highly associated with intercellular communication and brain disorders (Cheng et al., 2018).

Like ASD, a recent pathological study of the brains of schizophrenia (SCZ) patients found increased expression levels of TET1 mRNA and protein in the prefrontal cortex (PFC) (Dong et al., 2015). Consistent with this, a higher TET1 expression was found in hippocampus of SCZ mice model (Guidotti et al., 2014). The epigenetic profile of this model is characterized by a significant increase in TET1, and 5-hydroxymethylcytosine at SCZ candidate gene promoters and a reduction in the expression of glutamatergic and GABAergic genes (Guidotti et al., 2014).

## 2 Future directions

In this work, we have outlined a framework for understanding the mechanisms of neurological diseases through the lens of DNA and histone methylation. Our goal is to promote the development of innovative clinical applications by targeting epigenetic modifiers. Epigenetics provides an innovative and strong foundation for comprehending brain development, the generation of neural cellular diversity, the regulation of synaptic and neural network connectivity and plasticity, and the inheritance of complex cognitive and behavioral traits generations (Qureshi and Mehler, 2018; Ziffra et al., 2021). Recent analyses have identified characteristic combinations of phenotypes associated with NDD, linking them to specific molecular processes such as epigenetic regulation. In the future, cellular-resolution chromatin state datasets across various developmental and differentiation stages could provide a vital connection between mutations in chromatin modifiers and the selective vulnerabilities observed in different cell types of the developing human brain. These efforts will yield valuable insights into NDD pathology and improve predictive capabilities (Qureshi and Mehler, 2013).

Understanding the relevant molecular features of chromatin modifiers and what additional changes to chromatin allow active transcription of previously repressed genes will be essential to understanding the role of DNA and histone methylation in neural physiology and brain development. The partial redundancy observed among the chromatin modifiers in mammals makes it necessary to directly compare single, double, or even triple mutants of methyltransferases/demethylases to reveal the whole picture. This complexity poses significant challenges for experimental design and encourages further study of chromatin in neural physiology in model organisms with fewer chromatin-modifying enzymes. In line with this, organoids and assembloids could be used to study neurodevelopment by creating three-dimensional, self-organizing structures derived from patient-derived induced pluripotent stem cells (iPSCs), which mimic the developing human brain, allowing to investigate complex cellular interactions, regionalization, and neuronal connectivity in a controlled environment, particularly in the context of neurodevelopmental disorders and genetic mutations affecting brain development (Levy and Pasca, 2023). Further, pharmacological manipulation of chromatin modifiers in these settings can, in turn, bring insights into the pathophysiology of neurodevelopmental disorders.

In line with this, the success of HDAC inhibitors in clinics has propelled competition in the research on lysine demethylases (Duan et al., 2021; He et al., 2022). Several KDM inhibitors have been developed and are currently being tested in clinical trials to evaluate their safety and effectiveness. However, most of these trials focus on cancer. Interestingly, a brain-penetrant inhibitor of KDM1A was recently developed and tested in Alzheimer’s models. ORY-2001 improved memory deficit and behavior alterations in the senescence-accelerated mouse model and social avoidance in the rat-rearing isolation model. It has also been tested in a Phase IIa clinical trial for mild to moderate Alzheimer’s disease (Fang et al., 2019; Maes et al., 2020; Mehndiratta and Liou, 2020). It is possible that such novel epigenetics approaches of targeting chromatin modifiers directly may provide a more robust method of adjusting the synaptic activity and restoring brain connectome and could provide new avenues of treatments for patients with neurodevelopmental disorders.

It is important to exercise caution when considering the role of epigenetic mechanisms in NDDs. While epigenetic mechanisms such as histone modifications and DNA methylation have undoubtedly provided valuable insights into the regulation of gene expression in NDDs, it is essential to recognize that these processes alone do not fully explain the complexities of NDD pathophysiology. Epigenetics represents only one aspect of a broader regulatory network that governs gene expression, protein synthesis, protein activity, localization, and metabolic control—each crucial for neuronal development and function. Several intermediate steps exist between the genetic code and its ultimate influence on cellular behavior. For example, even if a gene is expressed, transcriptional regulation, RNA splicing, mRNA stability and transport, and protein translation initiation all significantly affect how effectively that gene is translated into functional proteins due to epigenetic modifications. Moreover, the localization of these proteins to specific cellular compartments, their post-translational modifications, and their interactions with other proteins or cellular structures further impact their activity and the overall cellular response. This cascade of events ultimately affects neuronal function, signaling, and plasticity, essential for brain development and function.

Moreover, metabolites can control pathways—such as regulating energy production, signaling molecules, and precursor availability—adding another layer of complexity that is often overlooked in epigenetic studies. These metabolic pathways can significantly influence how genetic information translates into biological function, affecting everything from protein synthesis to cellular homeostasis. Therefore, whether through altered nutrient sensing, mitochondrial dysfunction, or disrupted signaling, metabolic dysregulation may contribute to the neurodevelopmental deficits observed in these disorders, independent of or in conjunction with epigenetics changes. Additionally, many neurodevelopmental disorders involve complex multigenic and multifactorial interactions. This means it is not merely the modification of individual genes or chromatin states that leads to disease, but rather the interplay among genetic mutations, epigenetic alterations, protein dysfunction, cellular environments, and external factors such as stress or nutrition during critical developmental periods. The complexity of these interactions indicates that epigenetic mechanisms alone cannot fully explain the etiology or progression of neurodevelopmental disorders. Therefore, while epigenetic changes are essential for understanding specific aspects of neurodevelopmental disorders, it is crucial to view these mechanisms as part of a larger, more complex system that encompasses genetic, proteomic, and metabolic factors. Few examples underscore that while epigenetic mechanisms—such as DNA methylation and histone modifications—are essential in regulating gene expression and contribute to neurodevelopmental disorders, they do not fully explain the complex pathophysiology of these diseases. In many cases, the genetic mutations, absence of or dysfunction of protein, or toxic protein aggregation (e.g., Fragile X syndrome, HD) that occur in these disorders play a central role in the clinical phenotypes. Therefore, a comprehensive understanding of neurodevelopmental disorders must integrate epigenetic mechanisms and the broader genetic, proteomic, and cellular pathways that contribute to disease. An integrative approach that considers not only the regulation of gene expression but also the functional consequences of these changes at the protein, metabolic and cellular pathway levels will be essential for advancing our understanding of these intricate disorders and developing effective interventions and treatments.
